# Identification of Transcription Factor Binding Sites by Cleavage Under Target and Release Using Nuclease in Zebrafish

**DOI:** 10.1089/zeb.2021.0082

**Published:** 2022-06-10

**Authors:** Radwa Barakat, Clyde A. Campbell, Raquel Espin-Palazon

**Affiliations:** Department of Genetics, Development and Cell Biology, Iowa State University, Ames, Iowa, USA.

**Keywords:** CUT&RUN, zebrafish, protocol, transcription factor, chromatin profiling, epigenomics

## Abstract

Cleavage Under Targets and Release Using Nuclease (CUT&RUN) has emerged as a chromatin profiling strategy that excels traditional methods. Although CUT&RUN has been widely utilized in mammalian cells, its use in the zebrafish is at its early stages. In this study, we have developed a protocol to successfully perform CUT&RUN to map transcription factor (TF) binding sites in embryonic, adult tissues, and FACS-sorted zebrafish cells. We also provide a detailed workflow for the identification of predicted TF binding sites that can be utilized in any animal species. Altogether, our strategy will expand this invaluable tool to the zebrafish community, improving the epigenetic resolution that can be achieved in this model organism.

The zebrafish is an advantageous model organism to investigate *in vivo* gene function and tissue development.^1–4^ These depend on the spatiotemporal binding of transcription factors (TFs) to DNA enhancers and promoters that results in specific gene expression patterns. The ability to identify these interactions would greatly complement the advantages of this model organism. However, the zebrafish community has been unable to profile *in vivo* the epigenomic landscape of specific tissues because of the large quantity of cells needed to perform classical chromatin profiling techniques such as chromatin immunoprecipitation (ChIP).

Cleavage Under Targets and Release Using Nuclease (CUT&RUN) is a chromatin profiling strategy recently developed by Skene and Henikoff^5,6^ that overcomes the caveats of ChIP. It utilizes TF-specific antibodies that bind to a micrococcal nuclease to cleave the surrounding DNA. Cross-linkage with formaldehyde and solubilization are not necessary, increasing the resolution and reducing the artifacts and the number of cells needed.^5,6^ Although this technique has been used in yeast (*Saccharomyces cerevisiae*), *Drosophila melanogaster*, and mammalian cells,^5,6^ we have pioneered the use of CUT&RUN to map TF binding sites in the zebrafish.^7^ Our strategy for performing CUT&RUN in zebrafish will be described herein in high detail with the goal of expanding this epigenetic tool to the zebrafish community.

Our in-depth workflow for identifying *in silico* TF binding sites into a particular DNA sequence followed by empiric validation by CUT&RUN-qPCR in zebrafish cells is detailed in the Supplementary Data (Fig. 1). In addition, CUT&RUN-sequencing can also be performed after validation by CUT&RUN-qPCR. There are several situations, in which CUT&RUN-qPCR could be advantageous over CUT&RUN-sequencing, including (1) if the goal of the study is to identify if a TF binds a particular DNA locus, (2) to ensure that CUT&RUN was successful before spending costly resources and extended time performing sequencing, (3) CUT&RUN-qPCR could be done in most laboratories, however, CUT&RUN-seq requires specific sequencing technology and the ability to analyze next-generation sequencing (NGS) data, and (4) to quickly validate that an antibody not used before in CUT&RUN works on this application.

**FIG. 1.**
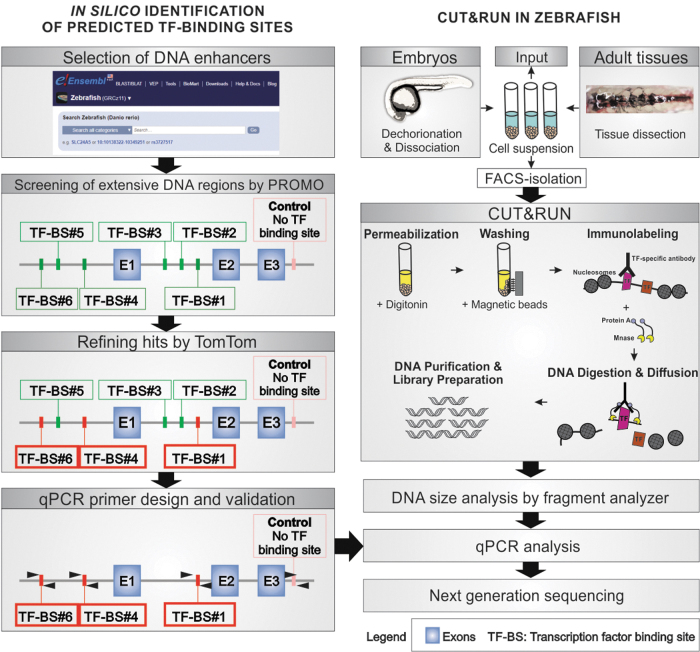
Schematic representation of workflow from the identification *in silico* of predicted transcription factor binding sites to its empiric validation by CUT&RUN. In brief, putative enhancer DNA sequences are identified by Ensembl.org, and analyzed by PROMO and TomTom. Predicted TF-BSs are utilized to design specific qPCR primers (*left panel*). Zebrafish embryos or adult tissues are dissociated and potentially sorted to generate a cell suspension that will undergo CUT&RUN, DNA size-, qPCR-, and NGS-analyzed (*right panel*). CUT&RUN, cleavage under target and release using nuclease; TF, transcription factor; BS, binding site; NGS, next-generation sequencing.

First, predicted binding sites (BSs) within regulatory DNA sequences for the desired TFs need to be identified. In brief, since proximal enhancers are typically located ∼5 kb from their paired promoter,^8^ 5 kb upstream and downstream the start codon of the gene of interest, as well as its intron sequences are identified in *ensembl.org*^9^ and analyzed by PROMO^10^ (Supplementary Fig. S1). The number of hits is then refined by TomTom^11^ (Fig. 1 and Supplementary Fig. S1). Using this workflow, we confirmed that 100% of the predicted BSs were indeed true BSs for p65 (*rela*) binding to *nfkbiaa* regulatory DNA elements (Supplementary Data).

Next, qPCR primers are designed flanking the TF predicted BSs. qPCR primer selection is critical. Although typically qPCR amplicon sizes for CUT&RUN- or ChiP-qPCR applications are 60–80 bp in length, we recommend reducing the amplicon size to 36–55 bp, since we have found that TFs in zebrafish typically yield DNA fragments <70bp after CUT&RUN (Supplementary Fig. S2 and Supplementary Data). The usage of primers with >60 bp amplicon can lead to false negative results.

A key step to successfully perform CUT&RUN is the ability to adequately dissociate the tissue in a short period of time with high cell viability. The protocol presented here is optimized to produce a cell suspension from zebrafish tissues in a rapid (<15 min) and efficient way (>99% survival) (Supplementary Fig. S3). To give the zebrafish community a broad array of tools for tissue dissociation, we provided here three dissociation protocols depending on the tissue of interest, including embryo dissociation, an example of zebrafish adult tissue (kidney marrow for hematopoietic studies) and FACS-sorted cells (Fig. 1 and Supplementary Data). This protocol will save operating time, and it ensures that the dissociated cells are intact for digitonin permeabilization.

On the contrary to ChIP applications performed in fixed cells, CUT&RUN utilizes live cells; thus, cell integrity and quality are maintained for optimal results. We have optimized this protocol for use with 20,000 to 100,000 zebrafish cells per sample.

After tissue dissociation, the cell suspension is ready to undergo CUT&RUN. We have obtained excellent results using the CUT&RUN Assay Kit by Cell Signaling (#86652), whose protocol was built based on the method protocol from Henikoff.^5,6^ We have followed the manufacturer's recommendations, with the following steps optimized for zebrafish. First, the cell suspension needs to undergo digitonin permeabilization. This essential step needs to be optimized for each cell type. Failure to do so will result in the inability of the antibody to diffuse into the cell and bind to its epitope, while also preventing the Micrococcal Nuclease (MNase) processed DNA fragments to freely exit the cell. We have determined that 24 hpf zebrafish embryos and adult tissue (kidney marrow) had an optimal performance for CUT&RUN using 2.5% digitonin. Antibodies were incubated over night at 4°C.

For the negative control, we recommend using 5 μL (dilution 1:20) of IgG isotype control antibody as recommended by the manufacturer (Cell Signaling; 86652S) provided by the kit as a negative control. Four microliters of p65 antibody (dilution 1:25) (PA5- 16545, ThermoFisher) was used per sample. Moreover, it is important to perform DNA purification using Phenol/Chloroform extraction according to CUT&RUN Assay Kit instructions to ensure the recovery of small DNA fragments typically yielded by TFs (Supplementary Fig. S2). Finally, an input DNA sample provides an additional negative control for CUT&RUN applications, reducing the potential bias generated by the IgG control,^12^ since the input sample provides a full representation of the cell genome.

In Supplementary Data, we have also included our optimized protocol to successfully obtain zebrafish chromatin fragments between 100 and 600 bp (Supplementary Fig. S4 and Supplementary Data).

CUT&RUN is revolutionizing our ability to profile the cellular epigenomic landscape with the highest resolution ever achieved. However, its implementation in the zebrafish model organism is just emerging, and an established protocol to successfully achieve CUT&RUN for TFs in zebrafish cells was missing. Here, we provide a detailed guide to translate this powerful technique for use in the zebrafish model. Included are protocols to efficiently dissociate zebrafish embryos and adult tissues, as well as a simple pipeline to identify potential TF BSs that can be validated by CUT&RUN-qPCR to ensure success before committing greater resources to NGS. Altogether, this protocol will facilitate the expansion of the epigenomic toolbox available to the zebrafish community.

## Supplementary Material

Supplemental data
