# Supplementary material for: Identification of Transcription Factor Binding Sites by Cleavage Under Target and Release Using Nuclease in Zebrafish
Source: Zebrafish. 2022 Jun 10;19(3):104–8. doi: 10.1089/zeb.2021.0082 (PMC9246268; doi:10.1089/zeb.2021.0082)
Supplement: Supplemental data [file Supp_Data.docx]

**Supplementary Data**

***In silico* detection of TF predicted binding sites**

Using our workflow described here (Fig. S1), we have previously demonstrated that Pu.1 (Spi-1 proto-oncogene b, encoded by *spi1b* gene) binds the regulatory DNA sequences of the granulin a (*grna*) gene^S1^. Later that year, others also reported the use of CUT&RUN in the zebrafish^S2, S3^. However, a detailed protocol to profile transcription factors in zebrafish by CUT&RUN is still missing. Here, as a proof of concept, we have expanded our analysis to an additional transcription factor, p65, to identify its potential binding sites for its known target gene *nfkbiaa*^S4-S6^. The workflow for this part is summarized in figure S1.

*Screening of extensive (~10,000 bp) DNA regions*

A 5 kb locus upstream the start codon, downstream the stop codon of *nfkbiaa*, as well as the intronic sequences were identified using <ensembl.org>^S7^. After selecting the species “*Danio rerio*”, search for the gene of interest and open the Transcript page. If more than one, select the accurate transcript of interest (gold transcripts are preferred since they are identical between Ensembl automated annotation and VEGA/Havana manual curation, and therefore they are stable). In the left-hand side bar click on” Exons” to find the first coding exon and initiation ATG. Click on “Configure page” on the left side. On “Flanking sequence at either end of transcript”, type in the number of bases pairs to show upstream and downstream (5,000 in our example). Also select “Show full intronic sequence” in the configuration tab. Save the selection by clicking on “Save and close” (the validation symbol √ at the upper right corner on the “Configure page” window). Copy your desired sequence in ApE or your preferred plasmid editor program.

Copy your sequence to be analyzed in PROMO^S8^ (http://alggen.lsi.upc.es/cgi-bin/promo_v3/promo/promoinit.cgi?dirDB=TF_8.3). We used the following PROMO features: “Select Species: All Species”, “Select Sites” (input the queried sequence), and “NF-kappaB T00591, T00590 and RelA T00594”. We found 8 unique predicted p65 BSs (Fig. S1). The putative BSs obtained by this method can be directly analyzed. However, we recommend to refine the *in silico* putative BSs using the motif comparison tool Tomtom^S9^ for more accurate results (see below).

Importantly, at least one DNA locus lacking predicted BSs for the TF of interest within the queried DNA must be identified, and primers designed, as a negative control. The Cq values resulting from the amplification of this “control locus” will be utilized for normalization against the queried putative BSs. In our example, we selected two fragments located at -5,717 and +5697, since they didn’t show any predicted binding sites for p65 (Fig. S1).

*Screening of short (40 bp) DNA regions*

It is common to find multiple (six or more) predicted BSs using PROMO. To circumvent this issue and restrict the search of potential BSs, an additional online tool can be utilized. We recommend using Tomtom^S9^ (<http://meme-suite.org/tools/tomtom>) to refine the search. Unfortunately, only small sequences (40bp or less) can be queried at a time using this tool. In our example, we selected 40 bp that included the predicted p65 binding motif found by PROMO within the middle of the 40 bp sequence. These were input within the “Input query motifs”. We utilized the settings enabled by default by the program, including “Eukaryote DNA” and “Vertebrates (*in vivo* and *in silico*)”. Following this workflow, five predicted binding sites were identified: BS1 (+830), BS2 (+895), BS3 (+1002), BS4 (+3078) and BS5 (+3264) (Fig. S1), and we subsequently proceed to the design of qPCR primers for empiric validation (see next section).

**Design of qPCR primers for the identification of predicted TF BSs**

qPCR primer selection is critical. For CUT&RUN-qPCR, primer length can vary from 18 to 22 bp, and the amplicon size should be approximately 36-55bp to avoid false negatives. It is important to keep in mind that, in contrast to ChIP, in which larger fragments are generated by sonication, the specific cleavage associated with CUT&RUN may sometimes generate fragments that are too small to amplify by qPCR (<20 bp). Primers should be designed with optimum melting temperature around 60°C, and 50-60% GC content. For each predicted binding site, primers were manually designed utilizing as DNA target the sequence containing the potential TF-BS as explained above. The GC-content, self-complementarity and the specific amplification across the genome were verified using Primer-BLAST^S10^ (<https://www.ncbi.nlm.nih.gov/tools/primer-blast/index.cgi>). Following current practices for qPCR primer validation as previously described^S11^ is also critical. List of primers used in current work is presented in the Supplementary Table 1.

**Zebrafish tissue dissociation**

*Whole zebrafish embryos & FACS-sorted cells*

Note: The protocol developed here is a modification and optimization from Svoboda and colleagues^S12^. Our dissociation protocol uses Liberase TM (Millipore-Sigma; REF: 5401127001), which contains highly purified Collagenase I and Collagenase II. We have found that the use of this purified collagenase blend maximizes viability and yield of isolated zebrafish cells over other methods, resulting in >99% viable cells (Fig. S3). Zebrafish embryos and adults were mated, staged, raised, and processed following the approved IACUC-20-025 and IACUC-20-024 protocols at Iowa State University.

1. Remove embryos from their chorion as previously described^S13^.

2. Transfer up to 50 zebrafish embryos to 1.5 ml tube. Wait 5-10 seconds until embryos deposit to the bottom by gravity.

3. Remove E2 embryo media. Avoid touching or damaging the embryos.

4. Add 500 µl per tube of PBS with Ca^2+^ Mg^2+^. Wait 5-10 seconds until embryos deposit to the bottom.

5. Remove PBS with Ca^2+^ Mg^2+^.

6. Add 500 µl per tube of Liberase TM (Roche, cat. no. 05401119001) at working solution (50 µg/ml in PBS with Ca^2+^ Mg^2+^). A stock 100x liberase TM can be prepared at 5 mg/ml by reconstituting the lyophilized enzyme with sterile water). Note: pre-warming the liberase TM working solution to 28.5ºC is recommended to increase its efficiency and reducing the dissociation time.

7. Gently perform an initial mechanical dissociation with a P1000 pipette set up to 400 µl (to avoid the introduction of bubbles) by gently pipetting up and down 5-10 times.

8. Incubate the tubes in a rotator at 28.5ºC 5 min for 24 hours post-fertilization (hpf) embryos, or 15 min for 48 hpf embryos.

9. Pipette the embryos gently up and down with a P1000 set up to 400 µl until the solution is homogeneous.

10. Transfer the solution to a 1ml syringe attached to a 20 gauge needle (Fisher Scientific; 148265C) containing a 30 µm nylon mesh (Fisher Scientific; NC9084441) between the syringe and the needle. Gentle push the plunger to filter the solution. Deposit the filtered solution into a flow cytometry tube.

11. Fill up the flow cytometry tube with 3 ml of cold PBS without Ca^2+^ Mg^2+^ (or cold FACS buffer, see below, for FACS applications).

12. Centrifuge the tubes at 300 rcf for 7 minutes at 4ºC.

13. Discard the supernatant by gently removing the liquid with a P1000 pipette. Keep the cells on ice from this point. Leave 150-200 µl of the solution at the bottom of the tube containing the cells. Discarding the supernatant by other methods such as decanting will considerably reduce the number of cells collected.

14. Resuspend the cells by gently pipetting the cells with a P1000. Avoid the introduction of air.

The cells are now ready for digitonin permeabilization and CUT&RUN.

Special notes for FACS applications:

Notes: to avoid cell breakage and death, it is critical to pipette cells with the largest volume pipette possible (P1000 preferred over P200, and this last over P10).

For FACS applications, wash with 3 ml of cold FACS buffer (0.9XPBS with 10% FBS) in step 10. Proceed to FACS by collecting the cells in 100 µl of 1X cold Wash Buffer containing Spermidine and Protease Inhibitor Cocktail as specified by the manufacturer in the CUT&RUN Assay kit (Cell signaling, 86652S). In our hands, this kit works efficiently and is user friendly.

We recommend 13 cycles during the PCR step during library preparation to reduce the duplication rates, resulting in cleaner NGS results.

Keep cells on ice until digitonin treatment.

After FACS, centrifuge at 300 rcf for 7 minutes at room temperature. Remove most of the supernatant, leaving 50 µl to avoid cell lost. Add 50 µl of fresh 1X Wash Buffer (+ spermidine + PIC) (total volume: 100 µl). Add 10 µl of activated bead suspension per reaction as indicated by the manufacturer CUT&RUN Assay kit (Cell signaling, 86652S) and follow steps in section II.

Using our method for embryo dissociation, we were able to obtain 30,000 cells per 24 hpf zebrafish embryo.

*Adult zebrafish tissues: kidney marrow*

Zebrafish are widely used for hematopoietic studies because of its high conservation to the human hematopoietic system^S14-S17^. As an example of adult tissue preparation for CUT&RUN, below is a protocol to prepare an optimized cell suspension from zebrafish kidney marrow cells, the hematopoietic organ in fish. Using the protocol described below, we found a survival rate greater than 99% (Fig. S3).

1. Dissect kidney marrow as described by Traver and colleagues^S18^. To eliminate mature erythrocytes that contaminate the sample from peripheral blood due to bleeding during the dissection, perform cardiocentesis as indicated^S18^.

2. Place kidney into 200 µl ice-cold 0.9× PBS containing 10% FBS.

3. Generate a single-cell suspension by gently pipetting tissue with a P1000 pipette (typically 10-20 times). The solution will become cloudy as the hematopoietic cells detach from the kidney.

Follow steps 9-13 in the “Whole zebrafish embryos & FACS-sorted cells” section above.

The obtained cell suspension is ready to use for digitonin treatment and CUT&RUN applications. With this dissociation method, 100,000 cells can be obtained per average per one kidney marrow.

**CUT&RUN**

In our hands, the CUT&RUN Assay Kit #86652 from Cell Signaling works efficiently for zebrafish cells. We followed the manufacturer recommendations with the following optimizations for zebrafish cells.

*Preparation of the Input Sample*

Input DNA sample provides an additional negative control for CUT&RUN applications. It may reduce the bias generated by the IgG control, since the input sample provides a full representation of the cell genome. It can be used for NGS after CUT&RUN and CUT&RUN-qPCR. As recommended by the manufacturer, sonication should be performed to obtain chromatin fragments between 100-600 bp. Below is an optimized protocol for 24 hpf zebrafish embryos.

1. Proceed to 24 hpf zebrafish cell dissociation as explained above. Collect 100,000 cells and centrifuged at 300 rcf for 7 minutes at room temperature.

2. Remove the supernatant and resuspend the cell pellet in 1 ml of Wash buffer.

3. Gentle pipette up and down to completely mix the cells with the buffer.

4. Centrifuge at 300 rcf for 7 minutes at room temperature. Remove the supernatant.

5. Wash the cell pellet by repeating steps 3 and 4.

6. Resuspend the cells in 100 µl of Wash buffer. Mix gently by pipetting.

7. Add 200 µl DNA extraction buffer and mix by pipetting.

8. Incubate at 55^o^C for 1 hour with shaking.

9. Place the tube on ice for 5 minutes to cool down.

10. Proceed to sonication to obtain DNA sizes between 100-600 bp. We tested 5, 10, and 15 sets of sonication cycles using Qsonica Q125 Sonicator Ultrasonic Homogenizer (Fig. S4A-C). The optimal sonication condition for zebrafish cells was 15 sets of 15 second pulses (Fig. S4C) using a 20 k HZ Sonicator with 1/8-inch probe, and incubating the samples for 30 seconds on ice between pulses.

11. Centrifuge at 18,500 rcf for 10 minutes at 4ºC to clarify the lysate. Transfer the supernatant to a new tube.

12. Purify the DNA using Phenol/Chloroform extraction and Ethanol precipitation as recommended by the manufacturer.

13. Determine DNA fragment sizes by electrophoresis or fragment analyzer.

*Digitonin permeabilization*

Notes: CUT&RUN relies on cell permeabilization by digitonin to allow for antibody penetrance and release of the digested DNA-TF-antibody complexes^S19^. Different cells present distinct sensitivity to digitonin, and therefore it is recommended to test the amount of digitonin needed to permeabilize >90% of the cells for each cell type as recommended by the manufacturer. We have used the CUT&RUN Assay kit, Cell signaling, 86652S and found that 2.5% digitonin works well for 24 hpf zebrafish embryos and adult zebrafish kidney marrow cells (data not shown).

*Antibody validation and empiric validation of TF binding sites*

Successful CUT&RUN requires good antibody reactivity. However, there is a reduced repertoire of available zebrafish-specific antibodies. To overcome this caveat, we propose to perform CUT&RUN pilot experiments to assess the cross reactivity in zebrafish for antibodies recognizing other species TFs. 70% of human genes have at least one obvious zebrafish orthologue, and zebrafish orthologous pairs are enriched in transcription factors^S20^. High identity among zebrafish and mammalian TFs is therefore often found, expanding the opportunities for the use of CUT&RUN in this model organism. We recommend to perform pilot CUT&RUN experiments to query the cross reactivity with zebrafish when utilizing an antibody that has not been validated in zebrafish. Since protein A binds to rabbit and guinea pig IgG but presents small affinity to mouse and goat IgG, a secondary antibody such as rabbit anti-mouse is advisable if using mouse or goat antibodies^S19^.

As a proof of concept, we demonstrated here that the mammalian-specific p65 antibody recognized its zebrafish ortholog.

1. Follow steps 1-13 from the “*Whole zebrafish embryos & FACS-sorted cells*” section to obtain a cell suspension from 24 hpf embryos.

2. Add 100,000-300,000 cells in 1.5 ml tubes, one for each antibody to be used. In addition to your experimental antibody condition, we recommend using 5 µl of IgG isotype control antibody as recommended by the manufacturer (Cell signaling, 86652S) and 4 µl of anti-zebrafish Pu.1 antibody (GTX128266, GeneTex)^S1^ as positive control for CUT&RUN of TF epigenomic profiling. The amount of antibody required for CUT&RUN differs and should be empirically determined by the user. Typically, between 1-9 µl per sample are required.

3. Proceed to digitonin permeabilization and CUT&RUN (see below).

4. After DNA purification by phenol/chlorophorm as indicated (Cell signaling, 86652S), proceed to analysis of DNA sizes by Fragment Analyzer or Bioanalyzer. A significant enrichment of small DNA fragments (<200 bp) should be observed in the experimental and positive control samples as compared to IgG control (Fig. S2). If this is observed, proceed to qPCR validation by following the workflow for the identification of BSs, qPCR primer design and validation as described here.

Library preparation is often not needed for an initial validation of the antibody by fragment analyzer if using a minimum of 100,000 cells, since small fragments will be abundant enough to be easily visualized. For CUT&RUN-qPCR applications, preparing a DNA library improves the detection of specific small DNA fragments. SimpleChIP ChIP-seq DNA Library Prep Kit for illumina (Cell Signaling, 56795S) with NEBNext Multiplex Oligos for Illumina 196 unique dual Index primers pairs (NEB, E6442S) can be utilized following the manufacturer modifications to enrich for small DNA fragments (Cell Signaling, 56795S). The presence of cleaved fragments and the size distribution should be evaluated via Byoanalyzer or TapeStation (Fig. S2). 5-10 million reads are sufficient for DNA sequencing.

As shown in Fig. S5, predicted p65 BSs 1, 2, 3, 4 and 5 showed statistically significant two- to eightfold enrichment compared to control isotype immunoglobulin G and internal control locus #1 and #2 (selected within the surrounding DNA loci that lacks predicted TF-BSs), demonstrating the successful recognition of the zebrafish ortholog p65 by the human antibodies utilized in CUT&RUN applications.

**FIGURE LEGENDS**

Figure 1. Schematic representation of workflow from the identification *in silico* of predicted transcription factor binding sites to its empiric validation by CUT&RUN. Briefly, putative enhancer DNA sequences are identified by Ensembl.org, and analyzed by PROMO and TomTom. Predicted TF-BSs are utilized to design specific qPCR primers (left panel). Zebrafish embryos or adult tissues are dissociated and potentially sorted to generate a cell suspension that will undergo CUT&RUN, DNA size-, qPCR- and NGS-analyzed (right panel).

Figure S1. Workflow showing the *in silico* identification of predicted p65 binding sites within the *nfkbiaa* regulatory DNA elements.





Figure S2. DNA size determination by Fragment Analyzer (AATI) showed that the use of the antibody that targets human versions of p65 in zebrafish cells yields the expected small DNA fragments (<100 bp, red square) that are reduced in an IgG control after performing CUT&RUN. LM, lower marker; UM, upper marker.





Figure S3. Cell viability after zebrafish embryo and kidney marrow dissociation. Representative flow cytometric side scatter and APC-A profile showing the percentage of live cells (Sytox Red^-^) from dissociated 24 hpf embryos (A) and adult zebrafish kidney marrow (B). The events shown were selected after doublet discrimination.





Figure S4. Input DNA size determination by Fragment Analyzer showing sonication conditions of 5 (A), 10 (B), and 15 sets (C) of sonication cycles using the Qsonica Q125 Sonicator Ultrasonic Homogenizer. The optimal sonication condition was 15 sets of 15 second pulses to obtain chromatin fragments between 100-600 bp. LM, lower marker; UM, upper marker.





Figure S5. P65 enrichment in *nfkbiaa* enhancers. CUT&RUN-qPCR was performed in 24 hpf zebrafish embryos using a p65 or control immunoglobulin G (IgG) antibody and two control loci that lacked predicted p65 BSs (located at -5,717 and +5697). Fold enrichment of p65-associated DNA fragments was identified by qPCR using primers flanking the BSs denoted in Fig. S1. To calculate the fold enrichment, qPCR results for each BS were normalized against spike-in DNA as described by the CUT&RUN Assay Kit #86652 (Cell Signaling), and control primers that amplify a locus of the *nfkbiaa* gene lacking predicted p65 BSs. This panel represents the average from four independent biological replicates performed. The primers used are shown in supplemental Table 1. Predicted p65 BSs 1, 2, 3, 4 and 5 showed statistically significant two- to eightfold enrichment compared to control isotype immunoglobulin G and internal control gene (*tert*),(*P≤0.05, **P≤0.01, ***P≤0.001). One-way ANOVA and Dunnett’s multiple comparisons test. Error bars represent Standard Error of the Mean (SEM).





Table 1. Primers utilized in this study.

| **Gene (locus)** | **Name** | **Nucleotide sequence (5’-3’)** |
| --- | --- | --- |
| *nfkbiaa*  Chromosome 20: 16,881,883-16,884,737 | nfkbiaaBS1F | GAAAACAAAGTGCGCAGGCA |
|  | nfkbiaaBS1R | CGCGCCAGGGACTTTCC |
|  | nfkbiaaBS2F | CCACACTGGCTGGAAATAACC |
|  | nfkbiaaBS2R | GCGTTTCGCAAGAATGGAGTTT |
|  | nfkbiaaBS3F | GCCTCCTACATGATTTTCCAGG |
|  | nfkbiaaBS3R | CGTGAATGATGGCAAGGTGC |
|  | nfkbiaaBS4F | GCAGGATTTGTGAATCGTAGGG |
|  | nfkbiaaBS4R | TGTCAAGAGAACAAAGAGATGGA |
|  | nfkbiaaBS5F | TGACTCAGTATAGAAAGTTCCCCAC |
|  | nfkbiaaBS5R | CAGCGGTGGTCTACTCATTTCT |
|  | ControlBS1F | CCTTAACATGCTGCAGTCCA |
|  | ControlBS1R | CACATTGTAGTAGTACTTTTAGTGG |
|  | ControlBS2F | AGCTTGAGGTCAGAAGAGAAAA |
|  | ControlBS2R | CCATCCATACAAACAATGTCCACC |

**Supplementary References**

S1. Campbell CA, Fursova O, Cheng X, et al. A zebrafish model of granulin deficiency reveals essential roles in myeloid cell differentiation. Blood Adv. 2021;5(3):796-811.

S2. Akdogan-Ozdilek B, Duval KL, Meng FW, Murphy PJ, Goll MG. Identification of chromatin states during zebrafish gastrulation using CUT&RUN and CUT&Tag. Dev Dyn. 2021.

S3. Ye Z, Braden CR, Wills A, Kimelman D. Identification of in vivo Hox13-binding sites reveals an essential locus controlling zebrafish brachyury expression. Development. 2021;148(11).

S4. Tian B, Nowak DE, Jamaluddin M, Wang S, Brasier AR. Identification of direct genomic targets downstream of the nuclear factor-kappaB transcription factor mediating tumor necrosis factor signaling. J Biol Chem. 2005;280(17):17435-17448.

S5. Brown K, Park S, Kanno T, Franzoso G, Siebenlist U. Mutual regulation of the transcriptional activator NF-kappa B and its inhibitor, I kappa B-alpha. Proc Natl Acad Sci U S A. 1993;90(6):2532-2536.

S6. Gao Z, Chiao P, Zhang X, et al. Coactivators and corepressors of NF-kappaB in IkappaB alpha gene promoter. J Biol Chem. 2005;280(22):21091-21098.

S7. Yates AD, Allen J, Amode RM, et al. Ensembl Genomes 2022: an expanding genome resource for non-vertebrates. Nucleic Acids Res. 2021.

S8. Messeguer X, Escudero R, Farre D, Nunez O, Martinez J, Alba MM. PROMO: detection of known transcription regulatory elements using species-tailored searches. Bioinformatics. 2002;18(2):333-334.

S9. Bailey TL, Boden M, Buske FA, et al. MEME SUITE: tools for motif discovery and searching. Nucleic Acids Res. 2009;37(Web Server issue):W202-208.

S10. Ye J, Coulouris G, Zaretskaya I, Cutcutache I, Rozen S, Madden TL. Primer-BLAST: a tool to design target-specific primers for polymerase chain reaction. BMC Bioinformatics. 2012;13:134.

S11. Bustin S, Huggett J. qPCR primer design revisited. Biomol Detect Quantif. 2017;14:19-28.

S12. Svoboda O, Stachura DL, Machonova O, Zon LI, Traver D, Bartunek P. Ex vivo tools for the clonal analysis of zebrafish hematopoiesis. Nat Protoc. 2016;11(5):1007-1020.

S13. Kim DH, Sun Y, Yun S, et al. Mechanical property characterization of the zebrafish embryo chorion. Conf Proc IEEE Eng Med Biol Soc. 2004;2004:5061-5064.

S14. Stachura DL, Traver D. Cellular dissection of zebrafish hematopoiesis. Methods Cell Biol. 2011;101:75-110.

S15. Espin-Palazon R, Weijts B, Mulero V, Traver D. Proinflammatory Signals as Fuel for the Fire of Hematopoietic Stem Cell Emergence. Trends Cell Biol. 2018;28(1):58-66.

S16. Davidson AJ, Zon LI. The 'definitive' (and 'primitive') guide to zebrafish hematopoiesis. Oncogene. 2004;23(43):7233-7246.

S17. Gore AV, Pillay LM, Venero Galanternik M, Weinstein BM. The zebrafish: A fintastic model for hematopoietic development and disease. Wiley Interdiscip Rev Dev Biol. 2018;7(3):e312.

S18. Traver D, Paw BH, Poss KD, Penberthy WT, Lin S, Zon LI. Transplantation and in vivo imaging of multilineage engraftment in zebrafish bloodless mutants. Nat Immunol. 2003;4(12):1238-1246.

S19. Skene PJ, Henikoff S. An efficient targeted nuclease strategy for high-resolution mapping of DNA binding sites. Elife. 2017;6.

S20. Howe K, Clark MD, Torroja CF, et al. The zebrafish reference genome sequence and its relationship to the human genome. Nature. 2013;496(7446):498-503.
